# Studying nursing activities in inpatient units: a road to sustainability for hospitals

**DOI:** 10.1186/s12912-022-00926-x

**Published:** 2022-06-13

**Authors:** Eman Kamel Hossny

**Affiliations:** Nursing Administration Department, Faculty of Nursing, Assiut University, Assiut city, 71526 Egypt

**Keywords:** Hospital sustainability, Inpatient units, Studying nursing activities

## Abstract

**Background:**

Since the United Nations has adopted the Sustainable Development Goals in 2015, sustainability has been increasingly considered. Working time is an important resource (time is money), as well as the nurses inside hospitals. So, nursing activities must be studied and analyzed well. Consequently, the resulting information gives hospital managers a clear picture of the current status—the basics of developing plans for sustainability and keeping pace with developed countries.

**Aim:**

This study was designed to study nursing activities performed in inpatient units and to determine which types of nursing activities are the most frequent and time-consuming, how much time each category of nursing personnel spends in different activities, and how units divide their time between patient care and other activities.

**Method:**

A work sampling method was used on 36 nurses in six units for three successive years, using two tools: a demographic data sheet and guidelines for the level of activities and area of activities.

**Results:**

There were 5,184 observations per year. According to area of activities, personal, and patient activities were the most time-consuming and frequent. According to the level of activities, unclassified and nursing activities were the most time-consuming among the intensive care, medical, and surgical units under study (44.1%, 41.6%, and 55.2%, respectively, and 28.2%, 34.8%, and 28.3%, respectively). The work of technical and diploma nurses was similar.

**Conclusion:**

Personal, unclassified, and patient activities consumed a large portion of nursing hours during the morning shift. Meanwhile, unit and personnel activities have consumed a minimal portion of hours. No significant differences in work were observed between technical and diploma nurses.

**Recommendations:**

Nursing managers and leaders should take a step to improve sustainability in their hospitals through study the nursing activities to gather data to develop plans for the future and rearrange the entire nursing staff in hospital units according to the needs of each shift.

## Introduction

Nowadays, hospital sustainability is a key concern for many hospital administrators. According to Lopes [1], management is the key to sustainability. The study of nursing activities within units in hospitals provides its managers with the necessary knowledge and facts about where nursing time is wasted. Accordingly, managers can take decisions and develop sound plans to avoid wasting work time and exploit the nursing workforce to the highest degree and take steady steps towards sustainability. Efforts lead to a sustainability that does not happen in hospitals without a long-term vision commitment and be reflected in the mission of the high-level organization [2].

This provides insight into the future of the nursing profession [3] and helps ourselves and our institutions prepare in this complex and fast economy, which usually provides superior opportunities for those who have forward access and deputy. According to Greek historians “Thucydides”, insisting on good knowledge of what happened in the past is the better way because similar events may occur in the future. Apt documents of nurses’ work is essential for decision support [4].

Although there remains major gaps in the evidence base on hospital sustainability, it is a wide-ranging and argued subject and often difficult to define; however, sustainability in the nursing context can be defined as follows: “Nurses partnering across geographical boundaries through cultural bridging, relationship and trust building, mutual goal setting, and evaluation of resource availability to raise the profile and support for nurses across regions. Equipped with new knowledge and skills, empowered nurses become change agents through education and training, leadership ventures, inter-professional bridging, and political advocacy, resulting in long-term capacity building, improved resource allocation, self-sufficiency, buy-in from local and national stakeholders, and a strengthened nursing workforce” [5].

Nurses are one of the main resources in healthcare settings, which comprise 60% of the healthcare system; in these settings, nurses should provide proficient, patient- centered, and cost-effective care. To do so, they must work at the top of their license. Therefore, it is the responsibilities of hospital managers to develop or rebuild the weaker portion inside their hospitals. In Egypt, hospitals are facing the phenomenon where nurses escape the profession [6]. Moreover, the world is facing nursing shortage, which has reached 20%. However, studies have indicated that staff shortage is considered an essential problem, which may negatively impact the quality of care and job satisfaction, and nurses spend a significant amount of non-valuable time on activities that can be eliminated while increasing cost-effectiveness [4].

There is a growing body of literature studying nurses’ work using observational studies; some studies have been conducted using electronic devices, such as a personal digital assistant, whereas others have used manual paper-based and stopwatch data collection [7].

Closely studying nursing activities allow to obtain accurate knowledge, which is the key to making good decisions [8], and allow nursing administrators in different healthcare organizations to understand well the nursing and non-nursing activities that consume a large portion of nurse’s time and to improve efficiency, workflow, and clinician job satisfaction [9].

Nursing activities refer to specific activities implemented by nurses to fulfill nursing procedures to promote patient’ improvement to anticipated results [10]. Nursing activities in any unit can be widely divided into direct patient care, indirect patient care, personnel, unit, and personal activities. Although each hospital is different, some problems are similar in all fields. If nurses spend long hours on personal matters instead of performing the intended activity, correcting this trend is necessary. Monitoring the activities from time to time between nurses can be performed [11]. Nursing activities must solve the problems that patients face. The quality of nursing activities should always be good to increase patient satisfaction, patient safety, and cost-effectiveness [12]. Nursing administrators and leaders play the largest role in solving those problems and moving toward sustainability.

By 2050, the need for nurses in healthcare will increase, especially with the current social, demographic, and epidemiological changes and the lack of global concern, which make healthcare more complex, because of the potential impact of nurses on access to care and quality [13, 14]. So, the ratio of nurses on a specific field, to the number of patients, must match the patients’ acuity level. Establish staffing levels that are flexible and accountable for changes is the responsibility of hospitals, according to ANA [15] that supports a legislative model in which nurses are empowered to create staffing plans specific to each unit. The normal ratio in inpatient units as (medical and surgical) 1: 6 and 1:2 in ICUs [16].

### Significance of the study

Sustainability occurs when resources are used as efficiently as possible, without compromising the quality of care for patients [14]; the results of the systematic review conducted by Cowie [15] revealed that the most frequently reported barrier to sustainability in hospitals was inadequate human resources in fifteen studies out of a total of thirty-two studies. Also, it was found the lack of time is an obstacle to sustainability in eight studies. In addition to the lack of awareness from the team of the problem. Moreover, it is well known that time and nursing personnel are one of the main resources inside the hospitals. Wasting nursing time in personal activities or unclassified activities related to nursing activities is considered a waste of working time, which cause the deterioration of the health services within those hospitals. It is the time for managers to look beyond the four walls of their organizations to have the knowledge related to nursing activities and know how nursing time is spent in patient rooms; studying nursing activities reveals to managers the truth of poor health services, dissatisfaction, and the deterioration of the health system within this hospitals which threatens its sustainability which requires drastic measures to avoid wasting working time in such activities. From a researcher’s viewpoint, it is the best approach for having best knowledge for solving the existing nursing problems inside each hospital. Therefore, the researcher conducted this study. The researcher selected the 6-h morning shift since a high volume of nursing activities is seen as representative during this time; moreover, during this time, most nursing activities are performed, the nurses’ work peaks, and different nursing categories are assigned to patient care.

### Study questions

1. What types of nursing activities are the most time-consuming and frequent?
2. Did the time spent by each nursing category in different activities differ?
3. How did the units under study divide their time between patient care and other activities?

### Aim

This study was designed to study nursing activities performed on inpatient units.

### Specific objectives

1. To determine types of activities that are the most time-consuming and frequent.
2. To determine how much time each category of nursing personnel spends on different activities.
3. To determine how units divided their time between patient care and other activities.

## Methods

### Design

A work sampling observational study was conducted over 6 days. This technique was first developed by Tippettin in 1935 cited by Munyisia [16] for use in industrial engineering and management. The researcher in this study adopted the manual “How to study nursing activities in a patient unit. This manual used a scientific method of studying activities of nursing personnel in hospitals, a method developed by the Division of Nursing Resources over a period of 4 years and carful tested in several leading hospitals. According to this method, the observer record what nurses on the unit does at 15 min intervals during the morning shift, in term of category of personnel performing the activity, the area into which the activity falls, and the level of personnel required for its performance.

### Study setting

The study was conducted in six units at a university hospital in Egypt, which have 1864 beds and 92 in-patient units. The six units were: two general medical units, two general surgical units, and two intensive care units. All six units with a total of 123 beds and 147 nurses (details in Table1). About the management, each unit contained a head nurse responsible for the main tasks of the nurses and a diverse number of nursing staff. All six units operate in a three-shift pattern: the morning shift from 7:30 a.m. to 1:30 p.m., the evening shift from 1:30 p.m. Until 7:30 p.m., the night shift is from 7:30 p.m. Until 7:30 a.m.

**Table 1:** Details of units under study

**Type of unit**	**No of beds**	**No of nurses**
General Surgical B1	23	15
General Surgical B2	23	13
Female general medical1	24	19
Female general medical 2	24	17
General intensive care	15	45
Emergency intensive care	14	38
Total	123	147

In this hospital, intensive care units were cardiac care unit (CCU) with 10 beds, general intensive care unit (ICU) with 15 beds, emergency ICU with 14 beds, chest ICU with 10 beds, cardiothoracic ICU with 8 beds and plastic ICU with 6 beds. General surgical units were surgical unit (A1, A2) composed of 7 rooms with 60 bed, surgical unit (B1, B2) 6 rooms with 46 beds and surgical unit (C1, C2) 4 room with 42 beds. General medical units were divided into male and female, female is 6 rooms with 48 beds and male is 8 rooms with 64 bed.

### Study participants

In this study, 36 nurses were enrolled: 16 from 2 medical units, 12 from 2 surgical units, and 8 from 2 intensive care units. In this research, technical nurses, as well as those with diplomas, were studied. The two categories have different educational levels; Technical nurses graduate after two years of study in nursing technical institutes after high school, and diploma nurses graduate after three years of nursing study after middle school.

#### Tool

The tool contains two parts. Part I was a demographic data sheet and was used to gather the demographic details of the participants, such as age, sex, educational qualification, experience, marital status, and workplace. Part II includes guidelines for level of activities and area of activities, which were developed based on the manual “How to study nursing activities in a patient unit.” The following levels of activities were evaluated: Administration (A) for instance (participate in a doctor's rounds without nursing intervention, and giving morning report to RN evening shift); Nursing (N) for instance (giving medications, dressing, Iv solutions therapy, and fix and change cannula and so on); Clerical (C) for instance (record request in patient's charts and recording requisition for medication); Dietary (D) for instance ( fed the patients); Housekeeping (H) for instance (clean drawers of the medication’s trolley); Messenger (M) for instance (Delivering medication requisition); and Unclassified (U) for instance (Conversation with her colleagues about personal affairs). The areas of activities were grouped into the following four activities: (1) patient: such as direct patient care and indirect patient care, (2) personnel: such as professional staff development, professional nursing student program, practical nursing student program, and other personnel activities, (3) unit: such as environmental, supplies and equipment, and other unit activities, and (4) personal: such as ‘personal telephone call’.

### Procedures

The research data were collected for three consecutive years, starting from the beginning of January to the middle part of February of 2017, 2018, and 2019. The actual time for collecting data in each year was approximately 6 weeks (one week for each unit). The data were obtained from work sampling records of what nursing personnel on the unit were doing during morning shifts. These records show the type of personnel observed (diploma nurses and technical institute nurses), the area of activity in which they are performing (shown in tool section), the level of skill required for the particular task, and a brief description of the activity.

The researcher arranged in advance with heads of the six nursing units that the morning shift’s nurses would continue working for 6 consecutive days. The observers used the pen and paper method; the data were collected by instantaneously observing the activities of nursing personnel on each unit for 15 min interval throughout the 6 h during morning shift (7:30 am to 1:30 pm).

Before the observation, the researcher trained two post graduate nurses (observers) by detailing the purpose, significance, study tools, clarify how to use the stopwatch, and explained the schedule of observations. The observers were asked to come to field 10 min before start the shift. Each observer started observations with the first person had meets with. The observer always watches, records each of the nurse’s activity, and records the taken time for finishing each activity. The observer attempted to observe different personnel in the same order each time. This method allows one’s different activities to be evaluated according to their run, duration, and frequency.

On each unit had been studied, a new series of observations started every 15 min. The first series started at a slightly different time each day for accurate investigate each activity. For example: Saturday’s observations on unit 1 started at 7 am, and later series of observations in the unit started 7:15 am, 7:30, 7:45, 8, and so on. On Sunday, observations started at 7:3 am, and continue at 7:18, 7:33, and so on. Monday’s observations then started at 7:6 am. The total number of observations for each nursing personnel for 6 days was 144 (24 observations each day).

### Statistical analysis

The recorded observations were tabulated manually in the following sequence: frequencies and percentages for qualitative variables. The observation records were recorded in order, and the total number of activities was counted without considering who performed those activities, the area, or the level (36 daily tally sheets) for all nurses. After that, the researcher first counted the total number of observations for all nurses on each day of observation in each unit (6 summary sheets), then counted the total number of observations for 6 days in each unit (6 summary sheets), and counted the grand total for all units for 6 days of observation (1 summary sheet for all days in all units). The total number of worksheets was 49. After these steps, analytical tables were prepared to determine what corrective actions were needed. In this study, along with the results of the 3 years of observation (Table 2), the researcher provided detailed results for a period of 1 year avoid repetition of data and not to overburden the reader.

**Table 2:** Demographic characteristics of the nursing personnel under study (*n* = 36)

**Demographic characteristics**	**No (%)**
**Age**	
Less than 30 yrs.	20 (55.6)
More than 30 yrs.	16 (44.4)
**Gender**	
Male	12 (33.3)
Female	24 (66.7)
**Education qualification**	
Secondary school of nursing diploma	24 (66.7)
Technical institute of nursing	12 (33.3)
**Experience**	
Less than 15 yrs.	21 (58.3)
More than 15 yrs.	15 (41.7)
**Marital status**	
Single	5 (13.9)
Married	31 (86.1)
**Units**	
ICU	8 (22.2)
Medical	16 (44.4)
Surgical	12 (33.3)

### Study terminologies

#### Definition of activity areas

Patient care: giving care means carrying out a nursing procedure for a patient or assisting doctors with treatments or procedures. Direct nursing care refers to activities in which the nurses spend time with the patient and/or the patient’s family; indirect nursing care refers to activities that are not applied directly to patients or are performed in preparation to engage in direct nursing care.

Personnel: in-service development of the staff refers to participations in activities which increase the knowledge and skills of personnel and thus contribute toward improved nursing service and better unit management.

Unit: refers to all units-centered activities including maintenance, cleanliness, orderliness, and safety; and issuance, maintenance, and requisitioning of supplies.

Personal: Personal activities refers to all activities that are personal, or to the time the individual is unoccupied and no unit purpose such as planning can be identified. Also refer to nonproductive activities, including inexorable delay and unnecessary rest time, drinking water, excretion, eating, personal telephone call, leaving the unit for personal affairs, and others.

Level of activity: refer to types of the tasks which differentiated on the basis of the degree and kind of skill, training, authority, and responsibility required to perform the activity successfully and efficiently. The levels of activity were coded as follows: Administration (A), Nursing (N), Dietary (D), Clerical (C), Housekeeping (H), Messenger (M), and Unclassified (U).

## Results

More than half (55.6%) of the nurses under study were less than 30 years old. Approximately two-thirds (66.7%) of them were female. Moreover, 66.7% of them graduated from secondary school of nursing diploma, whereas one-third (33.3%) of them graduated from technical institute of nursing. Approximately half (58.3%) of the nurses under study had less than 15 years of experience. Most (86.1%) nurses were married, and only 13.9% of them were single. Lastly, among all nurses who participated in this study, 44.5%, 33.3%, and 22.2% were assigned in the medical, surgical, and intensive care units, respectively (Table 1).

In the medical and intensive care units, over the 3 years, patient activities were the most time-consuming in the morning shift, followed by personal activities (292.4 and 236.4 h, 292.4 and 233.4 h, and 292.4 and 233.6 h, respectively, for the medical units and 133.2 and 126.6 h, 132.8 and 122.6 h, and 131.2 and 126 h, respectively, for the intensive care units). Meanwhile, in the surgical units, over the 3 years, personal activities were the most time-consuming in the morning shift, followed by patient activities (237.5 and 171 h, 233.5 and 171 h, 230.6 and 171 h, respectively). Additionally, unit and personnel activities had the least share of morning shift hours over the 3 years.

In the intensive care units under study, 46.3% of the work hours were spent on patient activities (directly and indirectly) (32.9% and 13.4%, respectively); most work hours were spent on providing patient care and exchanging information (18.4% and 8.9%, respectively). Then, 43.9% of the work hours were spent on personal activities, followed by unit activities (9.9%). Regarding unit activities, most work hours were spent on supplies and equipment and environmental activities (5.4% and 3.8%, respectively).

In the medical units, approximately half (50.7%) of the work hours were spent on patient activities. Most were spent on direct patient care (41.3%), mostly providing patient care and exchanging information (24.5%, and 11.8%, respectively). Then, 41% of the work hours were spent on personal activities. Then, 7.8% of the work hours were spent on unit activities, followed by environmental (4.2%) and personnel activities (0.5%).

**Fig. 1:**
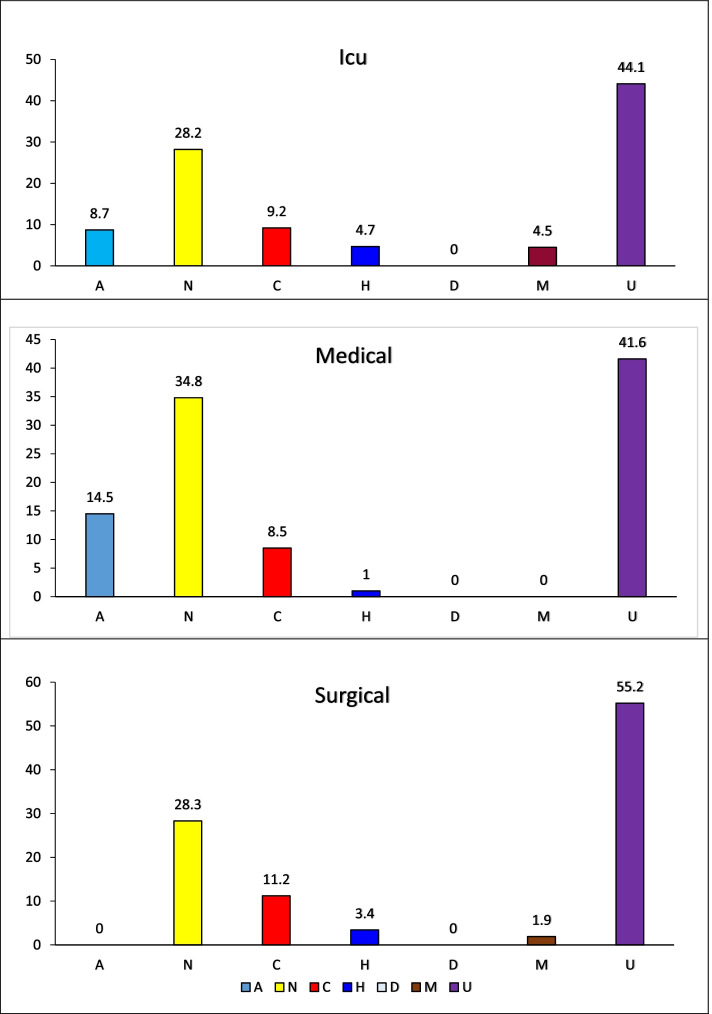
The percentage of work hours spent in the three units under study according to levels of activities

In the surgical units, more than half (55.2%) of the work hours were spent on personal activities. Then, more than one-third (39.6%) of the work hours were spent on patient activities, mostly direct and indirect patient care (26% and 13.5%, respectively). Lastly, 5.2% of the work hours were spent on unit activities (Fig. 1).

**Fig. 2:**
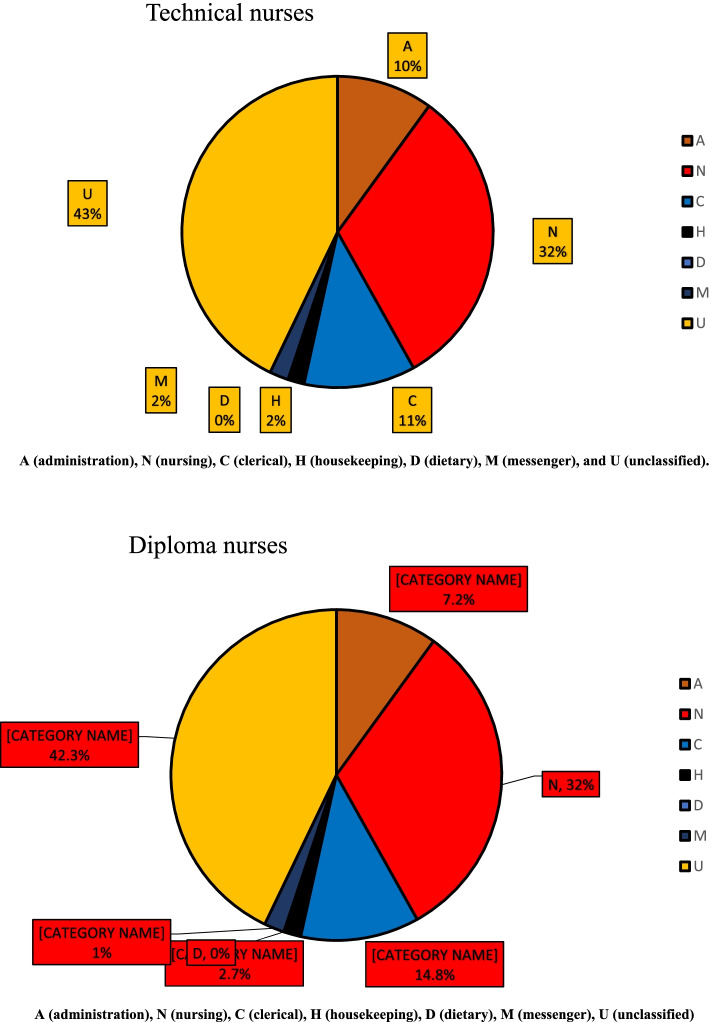
Percentage of work time spent by the nursing categories under study according to levels of activities

In the intensive care, medical, and surgical units under study, unclassified activities were the most time-consuming (44.1%, 41.6%, and 55.2%, respectively). Meanwhile, 28.2%, 34.8%, and 28.3% of the work hours, respectively, were spent on nursing activities. Approximately 9.2%, 8.5%, and 11.2% of the work hours in the intensive care, medical, and surgical units under study were spent on clerical activities, followed by administration (8.7%, 14.5%, and 0.0%, respectively), housekeeping (4.7%, 1.0%, and 3.4%, respectively), messenger (4.5%, 0.0%, and 1.9%, respectively), and dietary (0.0%, 0.0%, and 0.0%, respectively) activities (Fig. 2).

In the technical nursing category, approximately two-fifths (43.0%) of the work hours were spent on unclassified activities. Then, less than one-third (32.0%) of the work hours were spent on nursing activities, followed by clerical, administration, messenger, and housekeeping activities (11.0%, 10.0%, 2.0%, and 2.0% respectively).

**Fig. 3:**
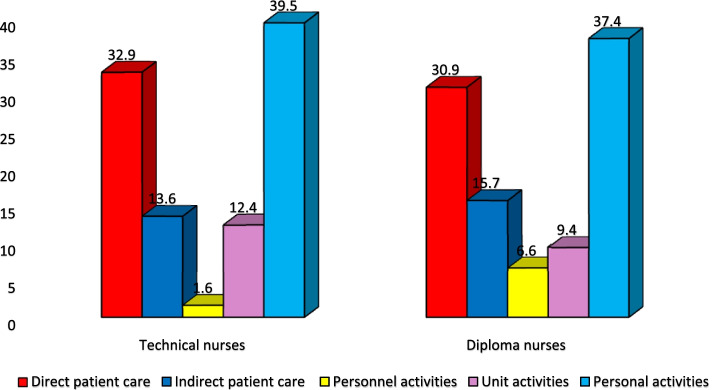
Percentage of work time spent by the nursing categories under study according to areas of activities

In the diploma nursing category, more than two-fifths (42.3%) of the work hours were spent on unclassified activities. Then, less than one-third (32.0%) of the work hours were spent on nursing activities, followed by clerical, administration, housekeeping, and messenger activities (14.8%, 7.2%, 2.7%, and 1.0%, respectively) (Fig. 3).

In the technical nursing category, the work hours were spent according to the following sequence: personal, direct patient care, indirect patient care, unit, and personnel activities (39.5%, 32.9%, 13.6%, 12.4%, and 1.6%, respectively).

In the diploma nursing category, their work time was spent according to the following sequence: personal, direct patient care, indirect patient care, unit, and personnel activities (37.4%, 30.9%, 15.7%, 9.4%, and 6.6%, respectively).

## Discussion

There were 5,184 observations for the nursing personnel under study for 6 h for six morning shifts over 3 successive years. This study examined nursing activities performed on inpatient units and determined which types of nursing activities are the most time-consuming and frequent, how much time each category of nursing personnel spends on different activities, and how units divided their time between patient care and other activities.

The data in Table 2 confirm more than two third of the nurses under study graduated from secondary school of nursing diploma. More than half of them were less than 30 years old and have less than 15 years of experience. The researcher followed the manner in which the working hours were consumed over 3 successive years (Table 3). A slight change was observed in the manner each unit spends their work time in each year, gearing toward patient, personal, unit, and personnel activities.

**Table 3:** The units under study according to areas of activity over 3 years

Areas of activities	1^st^ year			2^nd^ year			3^rd^ year		
	**ICU**	**Medical**	**Surgical**	**ICU**	**Medical**	**Surgical**	**ICU**	**Medical**	**Surgical**
**Total hours of all areas**	288 h	576 h	432 h	288 h	576 h	432 h	288 h	576 h	432 h
**Patient activities**	133.2	292.4	171	132.8	292.4	171	131.2	292.4	171
**Personnel activities**	0.0	3	0.0	8.3	6.2	0.0	5	10	5
**Unit activities**	28.6	45.2	23.5	24.3	44.0	27.5	25.8	40	26.4
**Personal activities**	126.6	236.4	237.5	122.6	233.4	233.5	126	233.6	230.6

In medical and intensive care units, over the 3 years of observation, patient activities especially direct patient care (productive) were the most time-consuming in the morning shift, followed by personal activities (non- productive) (near to half of total working h), whereas personal activities, followed by patient activities, were the most time-consuming in the surgical units under study. Meanwhile, unit and personnel activities were the least time-consuming and the least frequent. From the researcher’s viewpoint, nursing managers had a lack of knowledge about what happens inside their hospitals, leads to bad distribution of working hours on different activities within a nursing unit, and this has led to the overutilization of official working hours in nonproductive personal activities.

This suggests that the absence of control from managerial authoritarians, more problems are arising, and chances for sustainability are weak. A study in Egypt on declining employment in the nursing sector has revealed a phenomenon described as an escape outside the official healthcare system. This phenomenon was not a crisis resulting from a scarcity of specialization, but a crisis of poor distribution and the negative effects of policy-making priorities, which caused a shortage of nursing staff in hospitals [17].

Regarding the nursing categories (technical and diploma nurses), according to levels of activities (Fig. 2), both categories spent more than two fifth of work hours on unclassified activities (nonproductive), more than one third on nursing activities. They performed roughly similar activities with the same proportions without differentiating between academic qualifications, nursing skills, and experiences for each category during the morning shift, although most nursing activities were performed in this shift.

From another perspective, the data in Fig. 3 also confirm this result. When looking at the work hours of technical and diploma nurses according to areas of activities, it is observed that approximately two fifths of the work hours were spent on personal activities (nonproductive). Meanwhile, technical and diploma nurses spent about one third of the work hours on direct patient care. Also the least time for both categories were spent on personnel activities. From the researcher’s viewpoint, these results mean that both categories work the same tasks, have nearly the same amount of time, and perform the same activity classification. This indicates the absence of a methodology in distributing work for nursing categories. Equality in the distribution of work for the two categories of nursing, as they both have a different level of education, may cause disappointment in the higher category between them, and negatively affect their commitment to work, allowing the nursing staff to be lenient and disorganized.

According to the study by Neeraj [18], nurses working in an orthopedic ward of a tertiary care hospital in India spent 32% of their work time on nonproductive activities and 24% on patient care (basic and complex). Personal activities included off-station activities (24%), clerical activities (8%), administration activities (7%), supply and equipment maintenance (3%), and housekeeping activities (2%).

Regarding the work units (intensive care, medical, and surgical units) under study, despite differences in specialties among work areas, a slight difference in the manner they divided their work hours between patient care and other activities according to the area of activities (Table 4) and level of activities (Fig. 1); in intensive care units, 46.2% of total work hours were spent on patient care activities mostly in direct patient care (32%). Hence, this results support those of a preceding research performed in Finland [19]. In that study, nurses spend 38% of total nursing time on direct patient care, which is greater than the results of this study. Also in line with this results, the study conducted in China, the result found that ICU nurses spent most of their time in direct nursing care [20]. Followed by unit activities (9.9%). Personnel activities did not have any time, and 43.9% of the total work hours were spent on personal activities during the morning shift.

**Table 4:** Number and percentages of work hours in the three units under study according to areas of activities

Areas of activities	ICU No (%)	Medical No (%)	Surgical No (%)
Patient activities	133.2 (46.3)	292 (50.7)	171 (39.6)
Direct patient care	94.6 (32.9)	238.4 (41.3)	112.5 (26)
11. Giving care	53 (18.4)	141.2 (24.5)	97.5 (22.6)
12. Other direct activities	16 (5.6)	29.2 (5)	7.5 (1.7)
13. Exchange of information	25.6 (8.9)	68 (11.8)	7.5 (1.7)
Indirect patient care	38.6 (13.4)	54 (9.4)	58.5 (13.5)
14. Indirect care	38.6 (13.4)	54 (9.4)	58.5 (13.5)
Personnel activities	0.0 (0.0)	3 (0.5)	0.0 (0.0)
21. Professional staff development	0.0 (0.0)	0.0 (0.0)	0.0 (0.0)
22. Personnel: other	0.0 (0.0)	3 (0.5)	0.0 (0.0)
23. Professional nursing student program	0.0 (0.0)	0.0 (0.0)	0.0 (0.0)
24. Practical nursing student program	0.0 (0.0)	0.0 (0.0)	0.0 (0.0)
Unit activities	28.6 (9.9)	45.2 (7.8)	22.5 (5.2)
31. Environmental	11.0 (3.8)	24 (4.2)	13.5 (3.1)
32. Supplies and equipment	15.6 (5.4)	21.2 (3.6)	7.5 (1.7)
33. Other unit activities	2.0 (0.7)	0.0 (0.0)	1.5 (0.4)
Personal activities	126.6 (43.9)	236.4 (41)	238.5 (55.2)

In medical units, 50.7% of the total work hours during the morning shift were spent on patient care activities, 7.8% on unit activities, 0.5% on personnel activities, and 43.9% on personal activities. Meanwhile, in surgical units, 41.0% of the total work hours were spent on patient care activities, 5.2% on unit activities, and 55.2% on personal activities; personnel activities did not have any time. The results in Fig. 1 also confirm the same thing when looking at the division of work hours according to levels of activities in the units under study.

## Limitation of the work

There are some limitations regarding the generalizability of these results which are restricted to a small number of units, in the same hospital. Therefore, studying different nursing categories, different day shifts, different units and different hospitals will provide the desired data.

## Conclusion

This study is one of the few studies that have assessed nursing activities for three successive years, which provided real data on the number and percentages of working hours that are spent on various nursing activities in inpatient units. Based on the results of this study, less than half of the work hours over the 3 years were spent on nonproductive personal activities, and the remaining working hours were spent on patient, unit, and personnel activities. Personal or unclassified activities consume a large portion of nursing work hours during the morning shift. Moreover, patient activities consume a large portion, while other activities, especially unit and personnel activities, were allocated a minimal portion of hours during the morning shift. Regarding the technical and diploma nursing categories, both follow the same manner of spending working hours in inpatient units. A large part of their time was spent on personal activities, and the remaining hours were spent on direct and indirect patient care, unit activities, and employee activities.

## Recommendations

The researcher believes that the overall picture and the studied activities presented by the results of the study can lead the nursing managers to make the decision concerning morning shift dynamics so as to produce workflows that are confluent with nurses’ actual daily activities and work-load. Also, more attention is required from nursing managers in each unit regarding unit and personnel activities. Moreover, studying head nurse’s activity in each inpatient unit is recommended.

The managers should reconsider their hospitals and chaos to take the right step to sustainability. Studying nursing activities inside patients’ rooms enables nursing administrators and leaders to develop future plans and follow the pace of developed countries.

The results of this study predict that healthcare institutions will continue to suffer from nursing problems, such as nursing shortage and escape from the profession, unless formal nursing time is effectively used. Risks and opportunities do not await us; they will rush us when we are in the least prepared conditions, and subsequently, our only logical response is to think ahead and prepare ourselves as much as possible [21]. Therefore, nursing managers and leaders must study the nursing activities in their hospitals and rearrange the entire nursing staff in hospital units according to the needs of each shift. Furthermore, hospitals and other healthcare institutions should conduct training programs for nursing managers at all levels on how to study nursing activities.

Almost 100 years ago, Frederick Taylor argued that workers can be more productive if most activities were carefully studied to be more efficient with well-design and adequate motives for work performed. First-line nurse managers responsible for staff nurse activities must distribute activities on nursing personnel to match the shift hours using the job description of each category. Some nursing activities that consume the time of nurses should be reassigned to other personnel other than nurses whose time is allocated for patients, stressing on the importance of continuous supervision and monitoring for all nurses. Thus, nursing managers need to arrange shift dynamics to accommodate labor needs.

## Data Availability

All data generated and/or analyzed during the current study are not publicly available due the agreement between researcher and participants (privacy regulation) but are available from the corresponding author on reasonable request.
